# Reductive dissolution of As(V)-bearing Fe(III)-precipitates formed by Fe(II) oxidation in aqueous solutions

**DOI:** 10.1186/s12932-019-0062-2

**Published:** 2019-03-22

**Authors:** Andreas Voegelin, Anna-Caterina Senn, Ralf Kaegi, Stephan J. Hug

**Affiliations:** Eawag, Swiss Federal Institute of Aquatic Science and Technology, Ueberlandstrasse 133, 8600 Duebendorf, Switzerland

## Abstract

Iron(III)-precipitates formed by the oxidation of dissolved Fe(II) are important sorbents for major and trace elements in aquatic and terrestrial systems. Their reductive dissolution in turn may result in the release of associated elements. We examined the reductive dissolution kinetics of an environmentally relevant set of Fe(II)-derived arsenate-containing Fe(III)-precipitates whose structure as function of phosphate (P) and silicate (Si) content varied between poorly-crystalline lepidocrocite, amorphous Fe(III)-phosphate, and Si-containing ferrihydrite. The experiments were performed with 0.2–0.5 mM precipitate-Fe(III) using 10 mM Na-ascorbate as reductant, 5 mM bipyridine as Fe(II)-complexing ligand, and 10 mM MOPS/5 mM NaOH as pH 7.0 buffer. Times required for the dissolution of half of the precipitate (t_50%_) ranged from 1.5 to 39 h; spanning a factor 25 range. At loadings up to ~ 0.2 P/Fe (molar ratio), phosphate decreased the t_50%_ of Si-free precipitates, probably by reducing the crystallinity of lepidocrocite. The reductive dissolution of Fe(III)-phosphates formed at higher P/Fe ratios was again slower, possibly due to P-inhibited ascorbate binding to precipitate-Fe(III). The slowest reductive dissolution was observed for P-free Si-ferrihydrite with ~ 0.1 Si/Fe, suggesting that silicate binding and polymerization may reduce surface accessibility. The inhibiting effect of Si was reduced by phosphate. Dried-resuspended precipitates dissolved 1.0 to 1.8-times more slowly than precipitates that were kept wet after synthesis, most probably because drying enhanced nanoparticle aggregation. Variations in the reductive dissolution kinetics of Fe(II) oxidation products as reported from this study should be taken into account when addressing the impact of such precipitates on the environmental cycling of co-transformed nutrients and contaminants.

**Electronic supplementary material:**

The online version of this article (10.1186/s12932-019-0062-2) contains supplementary material, which is available to authorized users.

## Introduction

The oxidation of dissolved Fe(II) in aqueous solutions leads to the precipitation of amorphous to nanocrystalline Fe(III)-precipitates that critically impact on the cycling of nutrients and contaminants in aquatic and terrestrial systems [1, 2]. Examples include Fe(III)-precipitates forming at the redoxcline in lakes [3, 4], at anoxic groundwater exfiltration sites in streams and rivers [5–7] or in the marine environment [8] that control the cycling of phosphate, Fe(III)-precipitates accumulating in the rhizosphere of wetland plants that affect the uptake of As or other trace elements [9–11], or Fe(III)-precipitates forming in Fe-based techniques for As removal from drinking water [12–16] or for groundwater remediation and wastewater treatment [17–19].

The structure and composition of fresh Fe(III)-precipitates depend on the concentrations of solutes that interfere with Fe(III) polymerization and precipitation, including the oxyanions phosphate and silicate, Ca, Al, or dissolved organic matter [20–23]. The structure of Fe(III)-precipitates formed by dissolved Fe(II) oxidation at near-neutral pH in the presence of phosphate, silicate and Ca can be rationalized in terms of mixtures of three main structural endmembers: poorly-crystalline lepidocrocite, silicate-containing ferrihydrite, and amorphous Fe(III)-phosphate [20]. Variations in the structure of Fe(III)-precipitates between these endmember structures are expected to be reflected in variations in their biogeochemical reactivity, including their reductive dissolution kinetics.

In natural and engineered redox-dynamic systems, Fe(III)-precipitates formed by Fe(II) oxidation may again become exposed to anoxic conditions and undergo reductive dissolution. The reductive dissolution of Fe(III)-precipitates may lead to the release of nutrients and contaminants associated with the solids [24–26]. To assess the susceptibility of Fe(III)-precipitates to reductive dissolution and its impact on nutrient and contaminant cycling, knowledge on variations in the reductive dissolution kinetics of Fe(III)-precipitates as a function of precipitate composition and structure is needed. Reductive dissolution processes have been extensively studied for crystalline Fe(III)-((hydr)oxides) such as hematite, goethite and lepidocrocite as well as for synthetic 2-line ferrihydrite, which is considered a proxy for amorphous or poorly-crystalline Fe(III)-precipitates [27–30]. For 2-line ferrihydrite, also the impacts of precipitate freezing, drying and storage time on reductive dissolution rates have been examined [28]. However, synthetic 2-line ferrihydrite formed by the forced hydrolysis of a concentrated ferric iron solution may differ from Fe(III)-precipitates formed by the oxidation of dissolved Fe(II) at near-neutral pH in the presence of other solutes with respect to structure and reactivity. Accordingly, results gained on synthetic 2-line ferrihydrite alone do not allow to assess the variability of natural Fe(III)-precipitates in terms of structure and reactivity.

In recent work, we examined the variations in the composition and structure of Fe(III)-precipitates formed by the oxidation of dissolved Fe(II) in the presence of phosphate, silicate and Ca under conditions commonly observed in near-neutral natural (ground)waters [20]. Based on this work, the aim of the present study was to assess variations in the reductive dissolution kinetics of a representative set of Fe(III)-precipitates. For this purpose, reductive dissolution experiments were conducted in batch experiments with nine structurally different Fe(III)-precipitates in freshly synthesized (wet) form as well as after overnight drying and resuspension. Precipitate reduction kinetics were monitored in aerated suspensions containing 10 mM Na-ascorbate, 5 mM bipyridine (BPY), and 10 mM MOPS adjusted to pH 7.0. The role of BPY was to stabilize Fe(II) in the oxic solutions in dissolved form and to allow direct measurement of the formed Fe(II) by UV–Vis spectrometry. The total dissolved concentrations of Fe, P, Si and As were measured by inductively coupled plasma mass spectrometry (ICP-MS). The results were interpreted with respect to variations in the kinetics of precipitate dissolution and the congruence of P and As versus Fe release as a function of precipitate structure.

## Materials and methods

### Synthesis of precipitates

Precipitates for reductive dissolution experiments were prepared as described in previous work [20]. Briefly, background electrolyte was prepared by adding 4 mM CaCO_3_ or 8 mM NaHCO_3_ to doubly deionized (DI) water, purging with CO_2_ for ~ 5 min, stirring overnight to ensure complete dissolution (bottles closed with parafilm, pH ~ 5–6 in morning), adding Si (100 mM Na_2_SiO_3_ × 9H_2_O stock solution) to the (acidic) solution if required, raising the pH to 7.0 by purging with pressurized air, followed by addition of 7 µM arsenate (13 mM NaH_2_AsO_4_ × 7H_2_O stock solution). For each individual treatment, 800 mL of background electrolyte where then transferred to 1 L plastic flasks. Subsequently, phosphate was added as required (50 mM NaH_2_PO_4_ × H_2_O stock solution). Fe(III)-precipitate formation was initiated by adding 0.5 mM Fe(II) (50 mM Fe(II)SO_4_ × 7H_2_O stock solution acidified to pH ~ 3 with 1 mM HCl). After thorough shaking, 10 mL of unfiltered solution was collected and acidified with 0.65% HNO_3_ for analysis by inductively coupled plasma mass spectrometry (ICP-MS; Agilent 7500ce). The flasks were allowed to stand for 4 h, with hourly remixing. After 4 h, 10 mL of unfiltered suspension and 10 mL of filtered solution (0.1-µm cellulose nitrate membranes, 25 mm diameter) were acidified for analysis by ICP-MS. The pH was measured in the remaining suspension, before filtering twice about 400 mL of suspension through two filter membranes (0.1-µm cellulose nitrate membranes, 47 mm diameter). One of the membranes was dried overnight under a stream of pressurized air (dried precipitate), the other filter was stored overnight in a Petri disk wrapped with a moist tissue in a closed plastic box (wet precipitate). The day after synthesis, the dried and wet precipitates were scratched from the filter membranes, suspended in 1–2 mL DI water in Eppendorf tubes and dispersed in an ultrasound bath (4 min; 55 kHz, 19 W) for the subsequent dissolution experiments. All studied precipitates including their sample labels, synthesis conditions, and structural details (from previous work [20]) are listed in Table 1. The sample labels indicate the electrolyte cation (Ca or Na) as well as the initial phosphate/Fe(II) ratio (P/Fe)_init_ and the initial silicate/Fe(II) ratio (Si/Fe)_init_ (e.g., Ca-02-10 = precipitate synthesized by oxidation of 0.5 mM Fe(II) in 4 mM CaCO_3_ electrolyte at pH 7.0 at (P/Fe)_init_ = 0.2 and (Si/Fe)_init_ = 1.0).

**Table 1:** Precipitate synthesis, composition, and structure

Label	Synthesis solution^a^				Precipitate^b^			Structure^c^
	Cation	P/Fe (−)	Si/Fe (−)	As/Fe (−)	P/Fe (−)	Si/Fe (−)	As/Fe (−)	
Ca-00-00	Ca	−	−	0.014	−	−	0.013	100% pcLp
Ca-01-00	Ca	0.09	−	0.013	0.09	−	0.013	21% HFO; 79% pcLp
Ca-02-00	Ca	0.20	−	0.014	0.20	−	0.014	15% CaFeP, 26% HFO, 59% pcLp
Ca-05-00	Ca	0.53	−	0.016	0.53	−	0.016	59% CaFeP, 16% HFO, 25% pcLp
Ca-15-00	Ca	1.49	−	0.014	1.04	−	0.007	100% CaFeP
Na-15-00	Na	1.46	−	0.014	0.60	−	0.005	100% FeP
Ca-02-05	Ca	0.21	0.48	0.014	0.21	0.06	0.014	(25% CaFeP, 75% Fh-lowSi)
Ca-02-10	Ca	0.20	0.96	0.014	0.21	0.11	0.014	25% CaFeP, 75% Fh-Si
Ca-00-10	Ca	−	0.96	0.014	−	0.13	0.014	100% Fh-Si
2L-Fh^d^	Ca	−	−	−	−	−	−	2L-Fh

^a^Initial Fe(II) concentration 0.5 mM; pH 7.0 adjusted with CO_2_ in 4 mM CaCO_3_ (Ca) or 8 mM NaHCO_3_ (Na)

^b^Molar P/Fe and Si/Fe of precipitates calculated from difference between initial total and final dissolved P, Si and Fe concentrations measured by ICP-MS; values are average of triplicate samples (duplicate for Ca-00-00)

^c^Structure based on Fe K-edge EXAFS analysis of dried samples from Ref. [20] (*pcLp* poorly crystalline lepidocrocite, *HFO* hydrous ferric oxide, *CaFeP* amorphous Ca–Fe(III)-phosphate, *FeP* amorphous Fe(III)-phosphate), *Fh-Si* silicate-containing ferrihydrite. Precipitate Ca-02-05 has not been examined by EXAFS spectroscopy and the indicated structural composition is tentative (see text)

^d^2-line ferrihydrite synthesized by neutralization of 0.2 M Fe(NO_3_)_3_ × 9H_2_O with 1 M KOH [31]. Freshly prepared 2L-Fh was suspended in Ca electrolyte and wet and dry precipitates were prepared as for other Fe(III)-precipitates

An analogous reductive dissolution experiment was conducted with 2-line ferrihydrite (2L-Fh) synthesized by neutralization of 0.2 M Fe(NO_3_)_3_ × 9H_2_O with 1 M KOH [31]. Freshly prepared 2L-Fh was suspended in Ca electrolyte containing 7 µM As(V). Subsequently, wet and dried 2L-Fh samples were prepared for reductive dissolution experiments as described for the Fe(III)-precipitates.

### Reductive dissolution of precipitates

For the reductive dissolution experiments at neutral pH, a solution containing 10 mM MOPS (3-(*N*-morpholino)propanesulfonic acid) (pH buffer) and 10 mM Na-ascorbate (reductant) was adjusted to pH 7.0 by addition of ~ 5 mM NaOH, followed by the addition of 5 mM 2,2′-bipyridine [BPY, for Fe(II) complexation]. Ascorbic acid/ascorbate (pK_1_ = 4.25) has been extensively used to study the mechanisms and kinetics of reductive Fe(III)-(hydr)oxide dissolution and to reductively extract iron oxides from soils and sediments [27, 28, 32–37], but does not reduce sorbed As(V) [38]. BPY forms a very strong complex with Fe^2+^ (log β_3_ of 17.2 for Fe^2+^ + 3BPY = Fe(BPY)_3_^2+^ [39]) that can be used for the spectrometric quantification of Fe(II) [40]. BPY has previously been used to inhibit the oxidation of Fe^2+^ by O_2_ or H_2_O_2_ during the corrosion of zerovalent iron in oxic solutions [41, 42]. In the present study, the use of 5 mM BPY, in at least tenfold molar excess over total Fe, ensured a ratio of BPY-complexed over free Fe^2+^ of about 10^10^. At this ratio, BPY served to inhibit confounding reactions such as Fe(II)-induced transformation of the residual solids or precipitation of Fe(II)-phases, effectively stabilized Fe(II) against oxidation by O_2_, thus allowing to perform the experiments under oxic conditions, and enabled the direct determination of dissolved Fe(II) in filtered suspension aliquots using UV–Vis spectrometry.

For each experiment, the BPY solution was freshly prepared by dissolving BPY overnight under stirring in aluminum-wrapped glass bottles (Schott). For the dissolution experiments, 400 mL of the reaction solution was transferred into amber stained glass bottles (500 mL) and equilibrated in a water bath at 25 °C. The dissolution experiments were started by transferring the resuspended wet or dried precipitates into the 400 mL of reaction solution. During the experiment, the suspension was vigorously stirred to ensure representative sampling and the bottles were kept closed except for sample collection. In regular intervals, 5 mL of filtered solution (0.1-µm nylon membranes, 13 mm diameter) were collected for immediate analysis by ultraviolet–visible (UV–Vis) spectrometry for Fe(II) and by ICP-MS for total element concentrations (stored at 4 °C for ICP-MS analysis within 1 week). An unfiltered and a filtered sample were collected after the dissolution experiment and acidified with 1% HCl (v/v) for later analysis by ICP-MS. For most precipitates, the dissolution experiment was run in duplicate with about 12 h offset between the replicates, the second replicate serving mainly to cover the time period from ~ 12 to 24 h. Control experiments conducted in MOPS + BPY solutions without Na-ascorbate confirmed that BPY did not induce precipitate dissolution.

The analysis of the solution pH in some of the reductive dissolution experiments revealed that the pH gradually decreased over time to values as low as 6.7 within the 12-h time period. In experiments with slowly dissolving precipitates, the pH was observed to further decrease to values as low as pH 5.9 within 48 to 122 h. Without addition of a precipitate, on the other hand, the pH of the reaction solution remained close to 7.0 over time. A slight increase in pH would have been expected if OH^−^ released during reductive Fe(III)-precipitate dissolution had exhausted the buffer (which was not possible because the Fe concentration was at least 20 times lower than the buffer concentration). We speculate that the unexpected gradual decrease in pH over time may have been due to the oxidation of ascorbate to dehydroxyascorbate or another product with higher acidity (of the corresponding acid) or due to the partial decomposition of the buffer by reactive reaction intermediates. Since most of the kinetic data were derived from the first 12 h of the experiments or even shorter periods of time, the gradual decrease in pH over longer time periods was not considered to impact on the findings from this study.

### UV–Vis and ICP-MS analyses

The concentration of BPY-complexed Fe(II) in filtered solutions was derived from UV–Vis absorbance measurements at a wavelength of 522 nm right after sample collection (Cary 100, Varian Australia Pty Ltd.). For calibration, the absorbances of 0.01–0.1 mM Fe(II) were measured in undiluted MOPS/Na-ascorbate/BPY solutions and in solutions tenfold diluted with BPY-free MOPS/Na-ascorbate (pH 7.0) immediately after Fe(II) spiking as well as after 2 h reaction time. The results showed that neither sample dilution nor storage for 2 h substantially changed the absorbance at a given Fe(II) concentration (Additional file 1: Figure S1). From the calibration data, a molar extinction coefficient ε for the Fe(II)-BPY_3_ complex of ~ 8400 M^−1^ cm^−1^ at 522 nm was derived. For analysis, samples with less than ~ 0.1 mM Fe(II) (absorbance less than ~ 0.84 in 1-cm cuvettes) were measured without dilution, samples with higher Fe(II) concentrations after tenfold dilution in BPY-free MOPS/ascorbate solution adjusted to pH 7.0. Samples from the control experiments were collected and analyzed every 4 h.

The total concentrations of Na, Ca, Fe, P, Si, and As in acidified unfiltered and filtered samples collected during precipitate synthesis and dissolution were measured using ICP-MS, after dilution of the solutions with 0.65% HNO_3_. Total Fe in the dissolution experiments with the Fe(III)-precipitates ranged between ~ 0.2 and 0.5 mM; indicating that ~ 40–100% of the Fe used for precipitate synthesis was recovered after filtration of the synthesis suspension followed by sample recollection and resuspension. Total Fe concentrations in the experiments with 2L-Fh ranged between 0.05 and 0.2 mM (Additional file 1: Table S1).

## Results

### Composition and structure of the Fe(III) precipitates

The synthesis conditions and precipitate P/Fe and Si/Fe ratios [(P/Fe)_ppt_ and (Si/Fe)_ppt_, respectively] of the Fe(III)-precipitates used for this study are listed in Table 1. The measured precipitate P/Fe and Si/Fe ratios match with the results from our previous work on analogously synthesized samples, indicating that precipitate structure can be inferred from our earlier work in which we used X-ray absorption spectroscopy (XAS), X-ray diffraction (XRD), and transmission electron microscopy (TEM) for precipitate characterization [20]: Briefly, Precipitate Ca-15-00 formed at (P/Fe)_init_ of 1.5 in the absence of Si is an amorphous Ca–Fe(III)-phosphate. Precipitate Ca-00-00 formed in P-free solution corresponds to poorly crystalline lepidocrocite. Precipitates formed at (P/Fe)_init_ of 0.1 to 0.5 structurally correspond to mixtures of a decreasing fraction of poorly crystalline lepidocrocite and an increasing fraction of amorphous Ca–Fe(III)-phosphate. Precipitate Ca-00-10 formed in P-free solution at (Si/Fe)_init_ of 1.0 represents silicate-containing ferrihydrite with (Si/Fe)_ppt_ ~ 0.13. Structurally, the precipitate Ca-02-10 corresponds to a mixture of Ca–Fe(III)-phosphate and silicate-containing ferrihydrite [20]. The precipitate Ca-02-05 has been synthesized at an intermediate (Si/Fe)_init_ of 0.5. We have not analyzed the structure of this precipitate in our earlier study [20]. Considering that 0.5 (Si/Fe)_init_ have previously been shown to induce ferrihydrite formation and inhibit the precipitation of poorly-crystalline lepidocrocite during Fe(II) oxidation in synthetic groundwater [43], we expect this precipitate to structurally correspond to a mixture of amorphous Ca–Fe(III)-phosphate and Si-containing ferrihydrite with lower (Si/Fe)_ppt_ than in Ca-02-10. Precipitate Na-15-00 represents amorphous Fe(III)-phosphate. The lower (P/Fe)_ppt_ of the precipitate Na-15-00 than Ca-15-00 reflects that phosphate uptake in the Ca–Fe(III)-phosphate is enhanced by the formation of Ca–Fe(III)-phosphate and Ca-phosphate polymers in addition to Fe(III)-phosphate polymers [20].

### Precipitate reduction kinetics

**Fig. 1:**
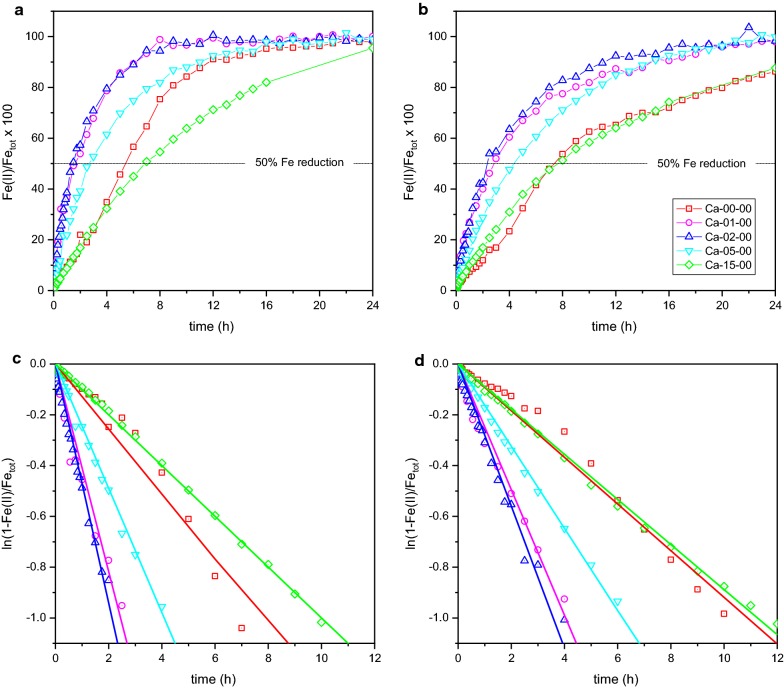
**a**, **b** Dissolved Fe(II) over the course of reductive precipitate dissolution for **a** wet and **b** dried precipitates formed by Fe(II) oxidation at (P/Fe)_init_ ratios from 0 (Ca-00-00) to 1.5 (Ca-15-00) in Ca electrolyte. Thin lines serve to guide the eye. **c**, **d** Corresponding plots of ln(1-Fe(II)/Fe_tot_), the natural logarithm of the residual Fe(III) fraction, versus time for **c** wet and **d** dried precipitates. Solid lines were calculated with k_app_ derived from the linear regression of the experimental data (Table 2)

**Fig. 2:**
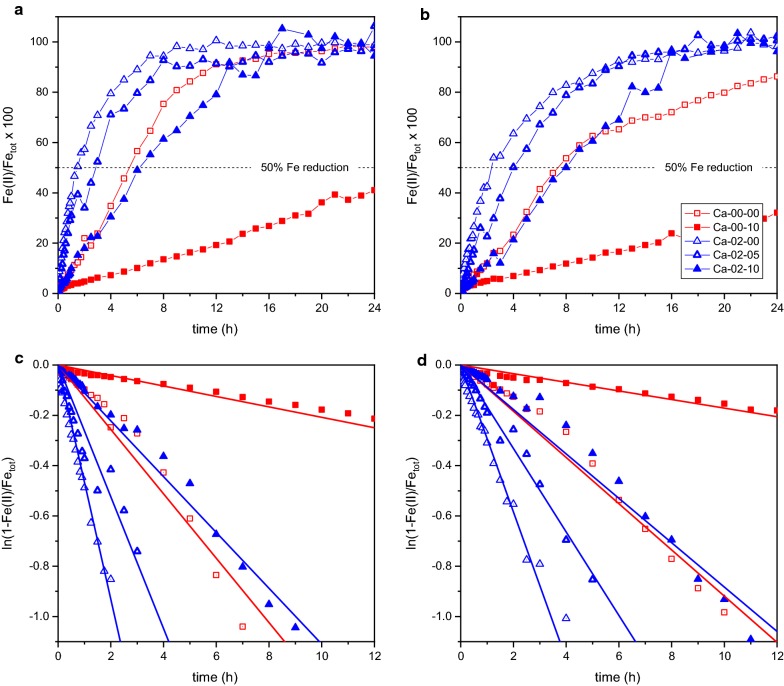
**a**, **b** Dissolved Fe(II) over course of reductive precipitate dissolution for **a** wet and **b** dried precipitates formed by Fe(II) oxidation at (P/Fe)_init_ of 0 (red curves) and (P/Fe)_init_ of 0.2 (blue curves) at (Si/Fe)_init_ of 0, 0.5 and 1.0. Thin lines serve to guide the eye. **c**, **d** Corresponding plots of ln(1-Fe(II)/Fe_tot_), the natural logarithm of the residual Fe(III) fraction, versus time for **c** wet and **d** dried precipitates. Solid lines were calculated with k_app_ derived from the linear regression of the experimental data (Table 2)

The increases in the fractions of dissolved (BPY-complexed) Fe(II) in the reductive dissolution experiments with precipitates formed in Ca-bicarbonate background electrolyte are displayed in Figs. 1a, b and 2a, b. For the precipitate Na-15-00 and for 2L-Fh, the data are displayed in Additional file 1: Figures S3ab and S4a in the additional file, respectively. For three treatments, comparison of dissolved Fe(II) determined by UV–Vis spectrometry and dissolved total Fe determined by ICP-MS confirmed that dissolved Fe essentially corresponded to Fe(II) (Additional file 1: Figure S2). Dissolved Fe(II) concentrations typically reached a plateau after a certain reaction time (Figs. 1a, b and 2a, b). The plateau concentrations of dissolved Fe(II) in general closely matched total Fe concentrations in unfiltered and filtered samples collected at the end of the experiments (Additional file 1: Table S1), indicating that the precipitates had completely dissolved. Accordingly, total Fe (Fe_tot_) derived from the last one to three UV–Vis measurements of the individual experiments together with the Fe(II) concentrations c(t) measured over the course of precipitate dissolution were used to calculate the fractions of dissolved Fe(II) (c(t)/Fe_tot_) and residual solid-phase Fe(III) (1 − c(t)/Fe_tot_) in the individual experiments (except for 2L-Fh, where Fe measured by ICP-MS in filtered samples was used for normalization, Additional file 1: Table S1).

The times required for the reductive dissolution of 50% of the total Fe (t_50 %_) in the individual experiments (Table 2) were derived from the fractions of dissolved Fe(II) shown in Figs. 1a, b, 2a, b, Additional file 1: Figures S3ab and S4a (either based on the data point at which 50% dissolution was observed, or by linear interpolation of the two data points adjacent to 50% dissolution). These t_50%_ values do not depend on the assumption of any specific rate law.

**Table 2:** Parameters of reductive dissolution kinetics

Label	Wet solids			Dried solids			Ratio dried/wet	
	t_50%_^a^ (h)	t_1/2_^b^ (h)	k_app_ (h^−1^)	t_50%_^a^ (h)	t_1/2_^b^ (h)	k_app_^c^ (h^−1^)	For t_50%_ (−)	For t_1/2_ (−)
Ca-00-00	5.4	5.4	0.13	7.4	7.5	0.092	1.36	1.39
Ca-01-00	1.6	1.7	0.41	2.8	2.8	0.25	1.78	1.67
Ca-02-00	1.5	1.5	0.47	2.3	2.5	0.28	1.55	1.70
Ca-05-00	2.7	2.8	0.25	4.3	4.3	0.16	1.63	1.55
Ca-15-00	6.9	6.9	0.10	7.6	7.8	0.089	1.11	1.12
Na-15-00	7.5	7.7	0.090	7.8	8.3	0.084	1.04	1.07
Ca-02-05	2.9	2.6	0.26	4.0	4.2	0.17	1.40	1.58
Ca-02-10	6.2	6.2	0.11	8.0	7.9	0.088	1.12	1.22
Ca-00-10	35	33	0.021	39	40	0.017	1.30	1.26
2L-Fh	5.0	5.6	0.12	27	−	−	5.33	−

^a^Time required for the dissolution of 50% of the solids; derived from Figs. 1a, b, 2a, b, Additional file 1: Figures S3ab, and S4a by interpolating data points adjacent to 50% Fe reduction or set to time at which 50% Fe reduction was measured

^b^Dissolution half-life time t_1/2_; calculated from k_app_ (t_1/2_ = ln(2)/k_app_)

^c^Apparent rate coefficient based on the assumption of pseudo-first-order dissolution kinetics; obtained by linear regression of linearized plots of ln(1 − c(t)/Fe_tot_) versus t for data points up to 63% Fe dissolution (Figs. 1c, d, 2c, d and Additional file 1: Figure S3cd). The relative standard error for k_app_ ranged from 0.5 to 5.5%

In Figs. 1c, d and 2c, d, linearized plots of the natural logarithm of the residual Fe(III) fractions (ln(1 − c(t)/Fe_tot_)) versus time are shown. In the case of pseudo first-order kinetics, these plots yield straight lines through the origin with a slope equal to the negative pseudo first-order rate coefficient k_app_ (ln(1 − c(t)/Fe_tot_) = − k_app_ × t). Apparent pseudo first-order rate coefficient k_app_ obtained from linear regressions limited to ln(1 − c(t)/Fe_tot_) from 0 to − 1 (i.e., up to ~ 63% precipitate dissolution) are listed in Table 2, the respective regression lines are shown in Figs. 1c, d, 2c, d and Additional file 1: Figure S3cd. The deviations of the experimental curves from the straight regression lines in some of the treatments indicated deviations from ideal pseudo-first-order dissolution kinetics. Precipitate half-life times t_1/2_ derived from the pseudo-first order rate coefficients k_app_ (t_1/2_ = ln(2)/k_app_) were very close to the times t_50%_ at which 50% of the precipitates had been dissolved (Table 2).

### Release of As and P versus Fe during reductive precipitate dissolution

**Fig. 3:**
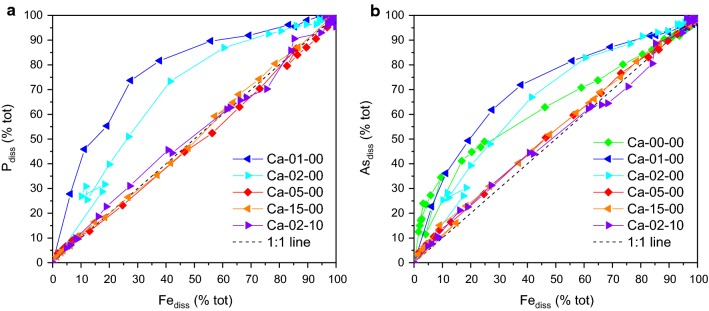
Dissolved fractions of **a** phosphate and **b** arsenate as a function of the dissolved fractions of Fe for wet precipitates. Data following the dashed 1:1 lines indicate congruent release of the respective oxyanion and Fe during reductive Fe(III)-precipitate dissolution, data above the 1:1 lines indicate preferential oxyanion release in the initial phase of reductive dissolution

For selected wet precipitates, the fractions of P or As(V) versus the fraction of Fe released over the course of Fe reduction are shown in Fig. 3. For the wet precipitates Ca-00-00, Ca-01-00, and Ca-02-00, containing a substantial fraction of poorly crystalline ferrihydrite, a preferential initial release of P (note: no P in Ca-00-00) and As(V) was observed. For the samples Ca-05-00 and Ca-15-00 dominated by amorphous Ca–Fe(III)-phosphate and the sample Ca-02-10 dominated by silicate-containing ferrihydrite and minor fraction of amorphous Ca–Fe(III)-phosphate, on the other hand, As(V) and P were released congruently with Fe.

## Discussion

### Reductive dissolution of wet and dried 2-line ferrihydrite

The reductive dissolution of 50% of the wet 2-line ferrihydrite prepared by forced hydrolysis of a concentrated Fe(III) solution according to a standard recipe in oxic 10 mM ascorbate/5 mM BPY solution at pH 7.0 took 5.0 h (Table 2). For analogously synthesized 2-line ferrihydrite, about 10–20 times shorter 50%-dissolution times have been observed in studies using deoxygenated 10 mM ascorbic acid solution at pH 3.0 [27, 30, 32] or deoxygenated 57 mM ascorbate/0.17 M citrate/0.6 M NaHCO_3_ solution at pH 7.5 [28] for reductive dissolution. The faster reduction in these earlier experiments may be attributed to the lower pH or the higher ascorbate concentration combined with citrate; or to the use of deoxygenated solutions, although BPY is expected to effectively stabilize Fe(II) in our experiments performed in non-deoxygenated solutions.

The dried ferrihydrite dissolved about five times more slowly than the wet ferrihydrite (Table 2), in line with previous studies reporting a marked decrease in the dissolution kinetics from fresh to dried 2-line ferrihydrite. This decrease has been attributed to precipitate aggregation during drying [28, 30] that may not be reversible during resuspension.

### Effect of drying on the reductive dissolution kinetics of Fe(III)-precipitates

**Fig. 4:**
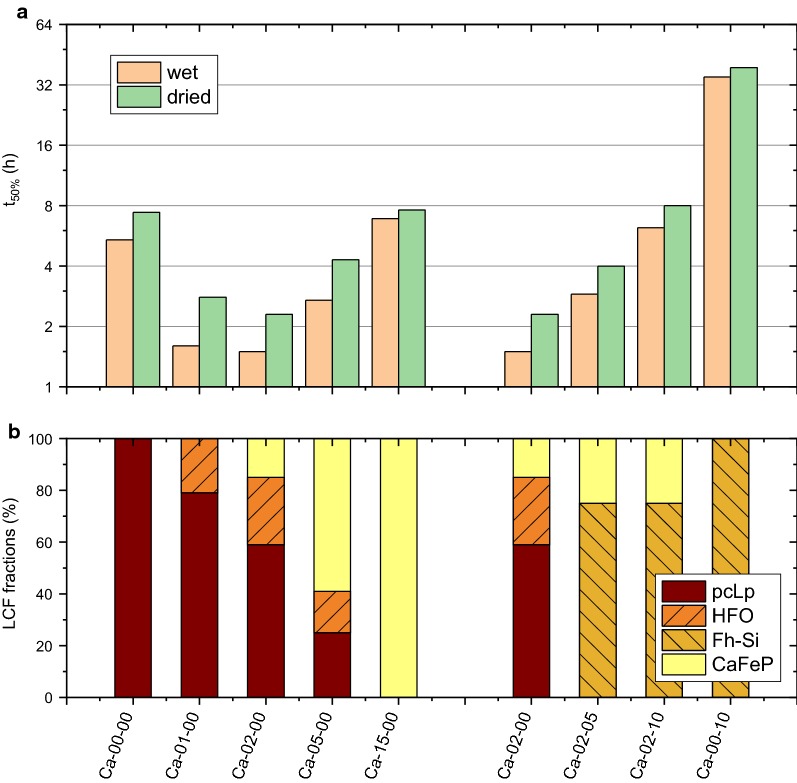
**a** Comparison of the times t_50%_ required for the dissolution of half of the Fe(III)-precipitate for selected wet and dried precipitates (values from Table 2). **b** Structural information obtained by Fe K-edge EXAFS spectroscopy on dried precipitates (from Ref. [20]; *pcLp* poorly-crystalline lepidocrocite; *HFO* hydrous ferric oxide, *Fh-Si* silicate-containing ferrihydrite, *CaFeP* amorphous Ca–Fe(III)-phosphate)

For the following discussion of the kinetics of the reductive dissolution of the Fe(II)-derived Fe(III)-precipitates and their link to precipitate composition and structure, the 50%-dissolution times t_50%_ determined in the present work for wet and dried precipitates and structural information from our previous study gained on dried precipitates [20] are summarized in Fig. 4. The t_50%_ of the dried precipitates were factor 1.04 to 1.78 (4 to 78%) higher than the t_50%_ of the respective wet precipitates, the t_1/2_ factor 1.07 to 1.70 higher (Table 2, Fig. 4). The drying-induced decrease in reduction kinetics was relatively small compared to variations in reduction kinetics induced by phosphate and silicate, and the dried precipitates still exhibited the same general trends in reduction kinetics as a function of phosphate or silicate as the wet precipitates (Table 2, Fig. 4a). This suggested that the structural changes induced by drying were relatively minor, and that the decrease in the reductive dissolution rates may have mainly been due to a drying-induced increase in nanoparticle aggregation that was not fully reversible during resuspenion.

Drying has a much stronger effect on the reductive dissolution kinetics of 2-line ferrihydrite synthesized by forced Fe(III) hydrolysis in the absence of P and Si, as observed in the present study and in previous work [28, 30]. This suggests that co-precipitated silicate or phosphate (Si-ferrihydrite, Fe(III)-phosphate) or the crystalline nature of the solid (poorly-crystalline lepidocrocite) limit the effect of drying on the reductive dissolution kinetics of Fe(III)-precipitates formed by the oxidation of dissolved Fe(II) in dilute aqueous solutions. The effect of drying on reductive dissolution kinetics, however, could not be clearly related to the composition or structure of the precipitates, although it seemed to be larger for more reactive precipitates; with the exception of P-free Si-ferrihydrite (Ca-00-10) and 2-line ferrihydrite (2L-Fh) (Table 2).

### Effect of phosphate on the reductive dissolution kinetics of Fe(III)-precipitates

The precipitates Ca-01-00 and Ca-02-00 formed at (P/Fe)_init_ of 0.1 and 0.2 dissolved about three times faster than P-free poorly-crystalline lepidocrocite (sample Ca-00-00; Table 2; Fig. 4). Considering that the samples Ca-01-00 and Ca-02-00 also contained a major fraction of lepidocrocite, this observation suggested that the increase in phosphate level led to the formation of even less crystalline and more reactive lepidocrocite. This observation is in line with a study on the effect of phosphate on lepidocrocite formation by Fe(II) oxidation [44]. In this study, phosphate at low levels around 0.03–0.05 (P/Fe)_init_ was shown to significantly decrease lepidocrocite crystallinity and to induce complete lepidocrocite solubility in acid oxalate solution (ligand- and proton-promoted dissolution). Amorphous Ca–Fe(III)-phosphate formed at a (P/Fe)_init_ of 1.5 (sample Ca-15-00) exhibited again a similar t_50%_ as the P-free poorly-crystalline lepidocrocite (sample Ca-00-00; Fig. 4, Table 2). The decrease in the reductive dissolution kinetics (increase in t_50%_) from Ca-02-00 to Ca-15-00 may be due to the extensive phosphate-coordination of oligomeric Fe(III) in the Ca–Fe(III)-phosphate, which may limit the formation of the Fe(III)-ascorbate complex required for reductive dissolution [36]. Ca-free amorphous Fe(III)-phosphate (sample Na-15-00) exhibited nearly the same dissolution kinetics as amorphous Ca–Fe(III)-phosphate (Table 2), suggesting that the enhanced polymerization of Fe(III) in Ca–Fe(III)-phosphate observed by XAS [20] did not inhibit reductive dissolution, although Ca leads to the stabilization of Fe(III)-phosphate with respect to precipitate transformation during aging [45].

Phosphate at a (P/Fe)_init_ of 0.2 not only increased the reductive dissolution kinetics of poorly-crystalline lepidocrocite (Ca-00-00 vs. Ca-02-00), but also significantly increased the dissolution kinetics of silicate-containing ferrihydrite (Ca-00-10 vs. Ca-02-10), to a level comparable to amorphous Ca–Fe(III)-phosphate (Ca-15-00) (Fig. 4, Table 2). Considering that arsenate has been reported to inhibit silicate polymerization on goethite at elevated loadings [46], we speculate that the effect of P on the dissolution kinetics of the Si-containing precipitates could be due to inhibited silicate sorption and polymerization (see next paragraph) in the presence of elevated levels of phosphate, which in turn could facilitate the access of ascorbate.

### Effect of silicate on the reductive dissolution kinetics of Fe(III)-precipitates

The wet Si-containing ferrihydrite precipitate Ca-00-10 dissolved ~ 7 times more slowly than wet poorly crystalline lepidocrocite (Ca-00-00) formed in P- and Si-free electrolyte (Table 2, Fig. 4). Furthermore, a clear increase in t_50%_ was also observed with increasing silicate level in the P-containing precipitates series Ca-02-00, Ca-02-05, Ca-02-10 (Fig. 4). Previous work indicated a ~ 2–3 times slower reductive dissolution of silicate-containing natural ferrihydrite (water treatment residues) [34] than of synthetic 2-line ferrihydrite in 10 mM ascorbic acid at pH 3.0 [27, 32], which has been attributed to a stabilizing effect of sorbed silicate [34]. We speculate that the pronounced inhibiting effect of silicate on reductive dissolution kinetics could be due to silicate binding and polymerization on the Fe(III)-precipitate surface [46–50] that might limit surface accessibility for ascorbate.

At the (Si/Fe)_init_ ratios of 0.5 and 1.0 examined in this study, Si induces the exclusive formation of Si-ferrihydrite in P-free solutions [43]. In previous work on the effect of Si on Fe(II)-derived Fe(III)-precipitates formed in P-free solutions [51], it has been found that increasing Si at very low (Si/Fe)_init_ ratios from 0.0007 to 0.050 led to the formation of decreasing fractions of lepidocrocite of decreasing crystallinity together with increasing fractions of Si-ferrihydrite. Considering the accelerating effect of P from 0 to ~ 0.2 (P/Fe) on reductive precipitate dissolution, which we attribute to a decreasing crystallinity of the lepidocrocite-type precipitate fraction, we speculate that increasing Si at very low Si/Fe ratios may have a similar effect on lepidocrocite crystallinity and reductive dissolution kinetics. At higher (Si/Fe)_init_ ratios as employed in the present study, on the other hand, the inhibiting effect of Si on the dissolution of Si-ferrihydrite prevails.

### Insights into precipitate structure from reductive dissolution experiments

In previous work, we characterized the structure of fresh Fe(III)-precipitates by XAS, X-XRD and TEM [20]. Based on XAS results, the precipitates studied in the present work were described as mixtures of the three endmember phases poorly-crystalline lepidocrocite (pcLp; precipitate Ca-00-00), amorphous Ca–Fe(III)-phosphate (CaFeP; precipitate Ca-15-00) and silicate-containing ferrihydrite (Si-Fh; precipitate Ca-00-10) as well as the intermediary phase hydrous ferric oxide (HFO) in Si-free electrolytes (Table 1, Fig. 4b). For phosphate-containing solutions at intermediate (P/Fe)_init_ ratios, we demonstrated that the formation of amorphous (Ca–)Fe(III)-phosphate preceded the formation of poorly-crystalline lepidocrocite (electrolytes without Si) or Si-containing ferrihydrite [electrolytes with (Si/Fe)_init_ of 1] [20, 52]. This sequential precipitate formation was reflected in the morphology of individual precipitate particles as revealed by TEM, which indicated the precipitation of lepidocrocite platelets on amorphous (Ca–)Fe(III)-phosphate in Si-free electrolytes [52, 53] and the formation of mixed Ca–Fe(III)-phosphate/Si-containing ferrihydrite particles in Si-containing electrolyte with a P-enriched core and a Si-enriched shell [20].

In the present work, complementary insight into precipitate structure and its impact on precipitate dissolution was gained from the congruence/incongruence of precipitate dissolution (Fig. 3) as well as from variations in the kinetics of precipitate dissolution between the different precipitates (Table 2, Fig. 4).

The precipitates Ca-00-00, Ca-01-00 and Ca-02-00 consisting of a major fraction of poorly-crystalline lepidocrocite (Table 1) showed a preferential initial release of phosphate and arsenate (Fig. 3), in line with the preferential release of As(V) during reductive dissolution of lepidocrocite by ascorbic acid at pH 3 reported in an earlier study [38]. Considering that the precipitates Ca-00-00 (poorly-crystalline lepidocrocite) and Ca-15-00 (amorphous Ca–Fe(III)-phosphate) exhibited similar dissolution kinetics, and that the precipitates Ca-01-00 and Ca-02-00 dissolved considerably faster (Table 2), the preferential initial release of phosphate and arsenate during the dissolution of the precipitates Ca-00-00, Ca-01-00 and Ca-02-00 was most probably due to the initial displacement of phosphate or arsenate adsorbed onto poorly-crystalline lepidocrocite by ascorbate, rather than due to a combination of preferential initial dissolution of As(V)- and P-containing Ca–Fe(III)-phosphate followed by slower dissolution of poorly-crystalline lepidocrocite. In contrast to the lepidocrocite-dominated precipitates, a congruent release of phosphate and arsenate with Fe was observed for the precipitates Ca-15-00 and Ca-05-00 dominated by amorphous Ca–Fe(III)-phosphate (Fig. 3, Table 1), indicating that the co-precipitated oxyanions were tightly bound in the precipitate structure.

Interestingly, also the precipitate Ca-02-10 which had previously been characterized as a mixture of 25% Ca–Fe(III)-phosphate and 75% silicate-containing ferrihydrite (Table 1) showed a congruent release of phosphate and arsenate with Fe, rather than preferential initial release of phosphate and arsenate, as could have been expected from the much faster reductive dissolution of pure Ca–Fe(III)-phosphate than silicate-containing ferrihydrite (precipitate Ca-15-00 versus Ca-00-10, Fig. 4). Similarly, also the dissolution kinetics of the precipitate Ca-02-10 provided no evidence for the presence of 25% rapidly-dissolving Ca–Fe(III)-phosphate and 75% slowly-dissolving Si-containing ferrihydrite (Fig. 2a, b). Thus, both the congruence and kinetics of the dissolution of the precipitate Ca-02-10 suggested that this precipitate reacted like a single phase whose bulk dissolution kinetics were accelerated by phosphate, rather than like a mixture of two separate phases. Considering that Ca–Fe(III)-phosphate polymers form first during Fe(II) oxidation [54, 55], we speculate that the aggregation of Ca–Fe(III)-polymers into precipitate nanoparticles is relatively slow due to their high negative surface charge [8] and that the aggregation of precipitate polymers into precipitate particles only becomes faster once larger and less negatively charged Si-ferrihydrite polymers form in the phosphate-depleted solution. As a result, individual precipitate nanoparticles may exhibit a gradual transition from a Ca–Fe(III)-phosphate-rich core to a Si-ferrihydrite-rich shell rather than a sharp core–shell separation, which may allow phosphate to accelerate the reductive dissolution of the entire precipitate.

### Environmental implications

In this study, we examined the reductive dissolution kinetics of a range of Fe(II)-derived Fe(III)-precipitates that are representative for Fe(III)-precipitates formed by the oxygenation of near-neutral natural waters, by the mixing of anoxic with oxic water, or at the redoxcline in a stationary water column. Our results on the dissolution kinetics of these amorphous to poorly-crystalline Fe(III)-precipitates show that the fastest dissolving solid (wet P-containing poorly-crystalline lepidocrocite; Ca-02-00) dissolved about 25 times faster than slowest dissolving solid (dried P-free Si-containing ferrihydrite; Ca-00-10) (Table 2, Fig. 4). This span in reductive dissolution kinetics can be explained by the effects of P, Si and drying: Low phosphate loadings (up to 0.2 P/Fe) increase the dissolution kinetics of the Fe(III)-precipitates by ~ 3–6 times relative to their phosphate-free counterparts, whereas higher phosphate loadings again decrease dissolution kinetics. Silicate loadings of ~ 0.1 Si/Fe on the other hand reduce the dissolution kinetics of P-free and P-containing Fe(III)-precipitates by a factor ~ 3–6. Relative to these variations related to phosphate and silicate and their impacts on precipitate structure, the slowing effect of drying on the dissolution of Fe(II)-derived Fe(III)-precipitates was less important (factor 1.0–1.8) and most probably related to enhanced nanoparticle aggregation.

The absolute reductive dissolution rates observed in this study are linked to the operationally defined reductive dissolution protocol and are therefore not directly transferable to natural environments. However, we postulate that the variations in reductive dissolution kinetics that we observed as a function of precipitate composition and structure—and hence as a function of Si/Fe and P/Fe ratios in the aqueous solutions from which they formed—are transferable to Fe(III)-precipitates with similar P/Fe and Si/Fe ratios formed by the oxidation of dissolved Fe(II) in natural waters at near-neutral pH.

Considering that Si/Fe ratios in natural water resources are often similar or even higher than the (Si/Fe)_init_ of 0.5 or 1.0 used to precipitate Si-ferrihydrite in this study [56, 57], and that natural Fe-precipitates or water treatment residues are often dominated by Si-ferrihydrite with Si/Fe ratios of 0.1 or higher [49, 56, 58], Si in many cases is expected to slow down the reductive dissolution of natural Si-ferrihydrite-rich Fe(III)-precipitates. With respect to the fate of co-precipitated As(V), the inhibiting effect of Si on reductive Fe(III)-precipitate dissolution and concomitant As(V) release parallels the inhibiting effect of Si on the structural transformation of Fe(III)-precipitates during aging and related As(V) release [45, 57]. Increasing phosphate loadings up to ~ 0.2 P/Fe, on the other hand, have an accelerating effect on the reductive dissolution kinetics of Fe(III)-precipitates dominated by Si-ferrihydrite and on the release of co-precipitated As(V); thereby contributing to the mobilizing effect of phosphate on As(V) that results from strong sorption competition between P and As(V) during both Fe(III)-precipitate formation and aging [15, 57].

## Conclusions

The results from this study emphasize that variations in the structure and composition of amorphous to poorly-crystalline Fe(III)-precipitates that are linked to their specific formation conditions can lead to substantial variations in their reactivity, as shown here with respect to reductive dissolution kinetics. Such variations should be taken into account when assessing the impacts of Fe(III)-precipitates on the fate of co-cycled nutrients and contaminants. Considering that, in addition to phosphate and silicate, also dissolved organic carbon may markedly affect the structure of Fe(III)-precipitates, further research is warranted on the coupled effects of inorganic and organic solutes on Fe(III)-precipitate formation, structure and reactivity. Finally, further research is needed to address variations in the structure and reactivity of amorphous or poorly-crystalline Fe(III)-precipitates formed by the neutralization of acidic Fe(III)-containing solutions in environmental systems.

## Additional file

**Additional file 1: Table S1.** Total added Fe in individual replicates as derived from Fe(II) and Fe(tot) in filtered and Fe(tot) in unfiltered samples collected at the end of each experiment. **Figure S1.** UV-Vis calibration data. **Figure S2.** Comparison of UV-Vis data for dissolved Fe(II) with ICP-MS data for total Fe in 0.1-µm filtered solutions from 3 dissolution experiments. **Figure S3.** Comparison of dissolution data of wet and dried amorphous Fe(III)-phosphate formed in the presence of Ca or Na. **Figure S4.** Dissolution data for fresh and dried 2-line ferrihydrite synthesized by forced hydrolysis of a concentrated ferric nitrate solution.

## Abbreviations

BPY: 2,2′-bipyridine
CaFeP: amorphous Ca–Fe(III)-phosphate
EXAFS: extended X-ray absorption fine structure
FeP: amorphous Fe(III)-phosphate
Fh: ferrihydrite
HFO: hydrous ferric oxide
ICP-MS: inductively coupled plasma mass spectrometer
MOPS: 3-(*N*-morpholino)propanesulfonic acid
pcLp: poorly-crystalline lepidocrocite
Si-Fh: silicate-containing ferrihydrite
TEM: transmission electron microscopy
UV–Vis: ultraviolet–visible
XAS: X-ray absorption spectroscopy
2L-Fh: 2-line ferrihydrite

## References

[1] Stumm W, Sulzberger B (1992). The cycling of iron in natural environments: considerations based on laboratory studies of heterogeneous redox processes. Geochim Cosmochim Acta.

[2] Taylor KG, Konhauser KO (2011). Iron in Earth surface systems: a major player in chemical and biological processes. Elements Mag.

[3] Cosmidis J, Benzerara K, Morin G, Busigny V, Lebeau O, Jézéquel D (2014). Biomineralizatin of iron-phosphates in the water column of Lake Pavin (Massif Central, France). Geochim Cosmochim Acta.

[4] Buffle J, De Vitre RR, Perret D, Leppard GG (1989). Physico-chemical characteristics of a colloidal iron phosphate species formed at the oxic-anoxic interface of a eutrophic lake. Geochim Cosmochim Acta.

[5] Baken S, Verbeeck M, Verheyen D, Diels J, Smolders E (2015). Phosphorous losses from agricultural land to natural waters are reduced by immobilization in iron-rich sediments of drainage ditches. Water Res.

[6] Fox LE (1989). A model for inorganic control of phosphate concentrations in river waters. Geochim Cosmochim Acta.

[7] Baken S, Moens C, van der Grift B, Smolders E (2016). Phosphate binding by natural iron-rich colloids in streams. Water Res.

[8] Gunnars A, Blomqvist S, Johansson P, Andersson C (2002). Formation of Fe(III) oxyhydroxide colloids in freshwater and brackish seawater, with incorporation of phosphate and calcium. Geochim Cosmochim Acta.

[9] Frommer J, Voegelin A, Dittmar J, Marcus MA, Kretzschmar R (2011). Biogeochemical processes and arsenic enrichment around rice roots in paddy soil: results from micro-focused X-ray spectroscopy. Eur J Soil Sci.

[10] Liu WJ, Zhu YG, Hu Y, Williams PN, Gault AG, Meharg AA (2006). Arsenic sequestration in iron plaque, its accumulation and speciation in mature rice plants (*Oryza sativa* L.). Environ Sci Technol..

[11] Hansel CM, Fendorf S, Sutton S, Newville M (2001). Characterization of Fe plaque and associated metals on the roots of mine-waste impacted aquatic plants. Environ Sci Technol.

[12] Meng X, Korfiatis GP, Christodoulatos C, Bang S (2001). Treatment of arsenic in Bangladesh well water using a household co-precipitation and filtration system. Water Res.

[13] van Genuchten CM, Peña J, Amrose SE, Gadgil AJ (2014). Structure of Fe(III) precipitates generated by the electrolytic dissolution of Fe(0) in the presence of groundwater ions. Geochim Cosmochim Acta.

[14] van Genuchten CM, Addy SEA, Pena J, Gadgil AJ (2012). Removing arsenic from synthetic groundwater with iron electrocoagulation: an Fe and As K-edge EXAFS study. Environ Sci Technol.

[15] Roberts LC, Hug SJ, Ruettimann T, Billah MM, Khan AW, Rahman MT (2004). Arsenic removal with iron(II) and iron(III) in waters with high silicate and phosphate concentrations. Environ Sci Technol.

[16] Voegelin A, Kaegi R, Berg M, Nitzsche KS, Kappler A, Lan VM (2014). Solid-phase characterization of an effective household sand filter for As, Fe and Mn removal from groundwater in Vietnam. Environ Chem.

[17] Furukawa Y, Kim J-W, Wilkin RT (2002). Formation of ferrihydrite and associated iron corrosion products in permeable reactive barriers of zero-valent iron. Environ Sci Technol.

[18] Fu F, Dionysiou DD, Liu H (2014). The use of zero-valent iron for groundwater remediation and wastewater treatment: a review. J Haz Mat..

[19] Wilfert P, Kumar PS, Korving L, Witkamp G-J (2015). The relevance of phosphorus and iron chemistry for the recovery of phosphorus from wastewater: a review. Environ Sci Technol.

[20] Senn A-C, Kaegi R, Hug SJ, Hering JG, Mangold S, Voegelin A (2015). Composition and structure of Fe(III)-precipitates formed by Fe(II) oxidation in near-neutral water: interdependent effects of phosphate, silicate and Ca. Geochim Cosmochim Acta.

[21] Adra A, Morin G, Ona-Nguema G, Menguy N, Maillot F, Casiot C (2013). Arsenic scavenging by aluminum-substituted ferrihydrites in a circumneutral pH river impacted by acid mine drainage. Environ Sci Technol.

[22] Mikutta C, Mikutta S, Bonneville S, Wagner F, Voegelin A, Christl I (2008). Synthetic coprecipitates of exopolysaccharides and ferrihydrite. Part I. Characterization. Geochim Cosmochim Acta..

[23] Eusterhues K, Wagner FE, Häusler W, Hanzlik M, Knicker H, Totsche KU (2008). Characterization of ferrihydrite-soil organic matter coprecipitates by X-ray diffraction and Mössbauer spectroscopy. Environ Sci Technol.

[24] Weber F-A, Hofacker A, Voegelin A, Kretzschmar R (2010). Temperature dependence and coupling of iron and arsenic reduction and release during flooding of a contaminated soil. Environ Sci Technol.

[25] Gächter R, Müller B (2003). Why the phosphorous retention of lakes does not necessarily depend on the oxygen supply to their sediment surface. Limnol Oceanogr.

[26] Grybos M, Davranche M, Gruau G, Petitjean P (2007). Is trace metal release in wetland soils controlled by organic matter mobility or Fe-oxyhydroxides reduction?. J Colloid Interface Sci.

[27] Larsen O, Postma D (2001). Kinetics of reductive bulk dissolution of lepidocrocite, ferrihydrite and goethite. Geochim Cosmochim Acta.

[28] Raiswell R, Vu HP, Brinza L, Benning LG (2010). The determination of labile Fe in ferrihydrite by ascorbic acid extraction: methodology, dissolution kinetics and loss of solubility with age and de-watering. Chem Geol.

[29] Cornell RM, Schwertmann U (2003). The iron oxides.

[30] Jones AM, Collins RN, Rose J, Waite TD (2009). The effect of silica and natural organic matter on the Fe(II)-catalysed transformation and reactivity of Fe(III) minerals. Geochim Cosmochim Acta.

[31] Schwertmann U, Cornell RM (1991). Iron oxides in the laboratory.

[32] Postma D (1993). The reactivity of iron oxides in sediments: a kinetic approach. Geochim Cosmochim Acta.

[33] Hyacinthe C, Bonneville S, Van Cappellen P (2006). Reactive iron(III) in sediments: chemical versus microbial extractions. Geochim Cosmochim Acta.

[34] Nielsen SS, Kjeldsen P, Hansen HCB, Jakobsen R (2014). Transformation of natural ferrihydrite aged in situ in As, Cr and Cu contaminated soil studied by reduction kinetics. Appl Geochem.

[35] Deng Y (1997). Effect of pH on the reductive dissolution rates of iron(III) hydroxide by ascorbate. Langmuir.

[36] Dos Santos Afonso M, Morando PJ, Blesa MA, Banwart S, Stumm W (1990). The reductive dissolution of iron oxides by ascorbate—the role of carboxylate anions in accelerating reductive dissolution. J Colloid Interface Sci.

[37] Suter D, Banwart S, Stumm W (1991). Dissolution of hydrous iron(III) oxides by reductive mechanisms. Langmuir.

[38] Pedersen HD, Postma D, Jakobsen R (2006). Release of arsenic associated with the reduction and transformation of iron oxides. Geochim Cosmochim Acta..

[39] Smith RM, Martell AE (1975). Critical stability constants.

[40] Moss ML, Mellon MG (1942). Colorimetric determination of iron with 2,2-bipyridil and with 2,2,2-terpyridil. Ind Eng Chem.

[41] Katsoyiannis IA, Ruettimann T, Hug SJ (2008). pH dependence of Fenton reagent generation and As(III) oxidation and removal by corrosion of zero valent iron in aerated water. Environ Sci Technol.

[42] Ling R, Chen JP, Shao J, Reinhard M (2018). Degradation of organic compounds during the corrosion of ZVI by hydrogen peroxide at neutral pH: kinetics, mechanisms and effect of corrosion promoting and inhibiting ions. Water Res.

[43] Schwertmann U, Carlson L, Fechter H (1984). Iron oxide formation in artificial ground waters. Schweiz Z Hydrol.

[44] Cumplido J, Barron V, Torrent J (2000). Effect of phosphate on the formation of nanophase lepidocrocite from Fe(II) sulfate. Clays Clay Min..

[45] Senn A-C, Kaegi R, Hug SJ, Hering JG, Mangold S, Voegelin A (2017). Effect of aging on the structure and phosphate retention of Fe(III)-precipitates formed by Fe(II) oxidation in water. Geochim Cosmochim Acta.

[46] Swedlund PJ, Din SU, Airey MAL, Kuo C, van de Weg AC, Vella JL (2015). A spectroscopic and Monte Carlo study of the unexpected promotion of interfacial H_4_SiO_4_ polymerization on an iron oxide in the presence of arsenate. Coll Surf A..

[47] Swedlund PJ, Sivaloganathan S, Miskelly GM, Waterhouse GIN (2011). Assessing the role of silicate polymerization on metal oxyhydroxide surfaces using X-ray photoelectron spectroscopy. Chem Geol.

[48] Parfitt RL, Van der Gaast SJ, Childs CW (1992). A structural model for natural siliceous ferrihydrite. Clays Clay Min..

[49] Carlson L, Schwertmann U (1981). Natural ferrihydrites in surface deposits from Finland and their association with silica. Geochim Cosmochim Acta.

[50] Cismasu AC, Michel FM, Tcaciuc AP, Brown GE (2014). Properties of impurity-bearing ferrihydrite III. Effects of Si on the structure of 2-line ferrihydrite. Geochim Cosmochim Acta..

[51] Schwertmann U, Thalmann H (1976). The influence of [Fe(II)], [Si], and pH on the formation of lepidocrocite and ferrihydrite during oxidation of aqueous FeCl_2_ solutions. Clay Miner.

[52] Voegelin A, Senn A-C, Hug SJ, Kaegi R. Dynamic Fe precipitate formation during Fe(II) oxidation in phosphate-containing aqueous solutions. In: Monte Verità Conference “Iron biogeochemistry—from molecular processes to global cycles”; 3–8. March 2013; Ascona, Switzerland; 2013

[53] Kaegi R, Voegelin A, Folini D, Hug SJ (2010). Effect of phosphate, silicate, and Ca on the morphology, structure and elemental composition of Fe(III)-precipitates formed in aerated Fe(II) and As(III) containing water. Geochim Cosmochim Acta.

[54] van der Grift B, Behrends T, Osté LA, Schot PP, Wassen MJ, Griffioen J (2016). Fe hydroxyphosphate precipitation and Fe(II) oxidation kinetics upton aeration of Fe(II) and phosphate-containing synthetic and natural solutions. Geochim Cosmochim Acta.

[55] Voegelin A, Senn A-C, Kaegi R, Hug SJ, Mangold S (2013). Dynamic Fe-precipitate formation induced by Fe(II) oxidation in aerated phosphate-containing water. Geochim Cosmochim Acta.

[56] Carlson L, Schwertmann U (1987). Iron and manganese oxides in Finnish ground water treatment plants. Water Res.

[57] Senn A-C, Kaegi R, Hug SJ, Hering JG, Voegelin A (2018). Arsenate co-precipitation with Fe(II) oxidation products and retention or release during precipitate aging. Water Res.

[58] Nielsen SS, Petersen LR, Kjeldsen P, Jakobsen R (2011). Amendment of arsenice and chromium polluted soil from wood preservation by iron residues from water treatment. Chemosphere.

