# Supplementary material for: Reductive dissolution of As(V)-bearing Fe(III)-precipitates formed by Fe(II) oxidation in aqueous solutions
Source: Geochem Trans. 2019 Mar 22;20:2. doi: 10.1186/s12932-019-0062-2 (PMC6430749; doi:10.1186/s12932-019-0062-2)
Supplement: Supplementary file 1 — Additional file 1: Table S1. Total added Fe in individual replicates as derived from Fe(II) and Fe(tot) in filtered and Fe(tot) in unfiltered samples collected at the end of each experiment. Figure S1. UV-Vis calibration data. Figure S2. Comparison of UV-Vis data for dissolved Fe(II) with ICP-MS data for total Fe in 0.1-µm filtered solutions from 3 dissolution experiments. Figure S3. Comparison of dissolution data of wet and dried amorphous Fe(III)-phosphate formed in the presence of Ca or Na. Figure S4. Dissolution data for fresh and dried 2-line ferrihydrite synthesized by forced hydrolysis of a concentrated ferric nitrate solution. [file 12932_2019_62_MOESM1_ESM.pdf]

Additional Information

Reductive dissolution of As(V)-bearing Fe(III)-precipitates formed by Fe(II) oxidation in aqueous solutions

Andreas Voegelin,[*] Anna-Caterina Senn[1], Ralf Kaegi[1], Stephan J. Hug[1]

[1] Eawag, Swiss Federal Institute of Aquatic Science and Technology, Ueberlandstrasse 133, CH-8600 Duebendorf, Switzerland

[*] Correspondence: andreas.voegelin@eawag.ch

(1 table, 4 figures)

Table S1. Total added Fe in dissolution experiments, inferred from Fe(II) (from UV-Vis) and Fe(tot) (from ICP-MS) in filtered samples and Fe(tot) in unfiltered samples collected at the end of the dissolution experiments. Values in bold were used to normalize dissolved Fe(II) concentrations measured over the course of the dissolution experiments.

**Sample**	**Replicate**	**Fe(II) (filt)[a]**	**Fe(tot) (filt)**	**Fe(tot) (unfilt)**	**0-12 h[b]**	**end time[c]**
		**(mM)**	**(mM)**	**(mM)**		**(h)**
**Ca-00-00**	wet 1	**0.39**	0.39	0.39	x	53
	dried 1	**0.38**	0.38	0.40	x	53
**Ca-00-01**	wet 1	**0.43**	0.43	0.44	x	36
	wet 2	**0.46**	0.46	0.45		24
	dried 1	**0.45**	0.45	0.44	x	36
	dried 2	**0.42**	0.42	0.45		24
**Ca-00-02**	wet 1	**0.44**	0.44	0.43	x	50
	wet 2	**0.43**	0.43	0.43		40
	dried 1	**0.42**	0.42	0.42	x	50
	dried 2	**0.41**	0.41	0.41		40
**Ca-00-05**	wet 1	**0.38**	0.38	0.38	x	52
	wet 2	**0.44**	0.44	0.44		42
	dried 1	**0.42**	0.42	0.43	x	52
	dried 2	**0.41**	0.41	0.43		42
**Ca-00-15**	wet 1	**0.47**	0.47	0.47	x	50
	wet 2	**0.37**	0.37	0.38		38
	dried 1	**0.51**	0.51	0.50	x	50
	dried 2	**0.43**	0.43	0.43		38
**Na-00-15**	wet 1	**0.24**	0.24	0.24	x	50
	wet 2	**0.24**	0.24	0.24		40
	dried 1	**0.19**	0.19	0.20	x	50
	dried 2	**0.24**	0.24	0.24		40
**Ca-10-00**	wet 1	**0.31**	0.31	-[d]	x	122
	wet 2	**0.28**	0.28	-[d]		112
	dried 1	**0.28**	0.28	-[d]	x	122
	dried 2	**0.24**	0.24	-[d]		112
**Ca-05-02**	wet 1	**0.27**	0.27	0.40	x	36
	wet 2	**0.34**	0.34	0.35		24
	dried 1	**0.40**	0.40	0.27	x	36
	dried 2	**0.39**	0.39	0.40		24
**Ca-10-02**	wet 1	**0.39**	0.39	0.39	x	52
	wet 2	**0.39**	0.39	0.41		40
	dried 1	**0.41**	0.41	-[d]	x	52
	dried 2	**0.39**	0.39	0.41		40
**2L-Fh**	wet 1	0.19	**0.18**	0.17	x	50
	wet 2	0.15	**0.15**	0.13		38
	dried 1	0.11	**0.11**	0.10	x	50
	dried 2	0.04	**0.05**	0.04		38

[a] In four experiments, the analysis of two or three samples collected at the end of the experiment returned a relative standard deviation of at most 5%.
[b] treatment that covered period 0-12 h (and later periods starting from 24 h)
[c] time the last sample was collected
[d] erroneous or missing measurement

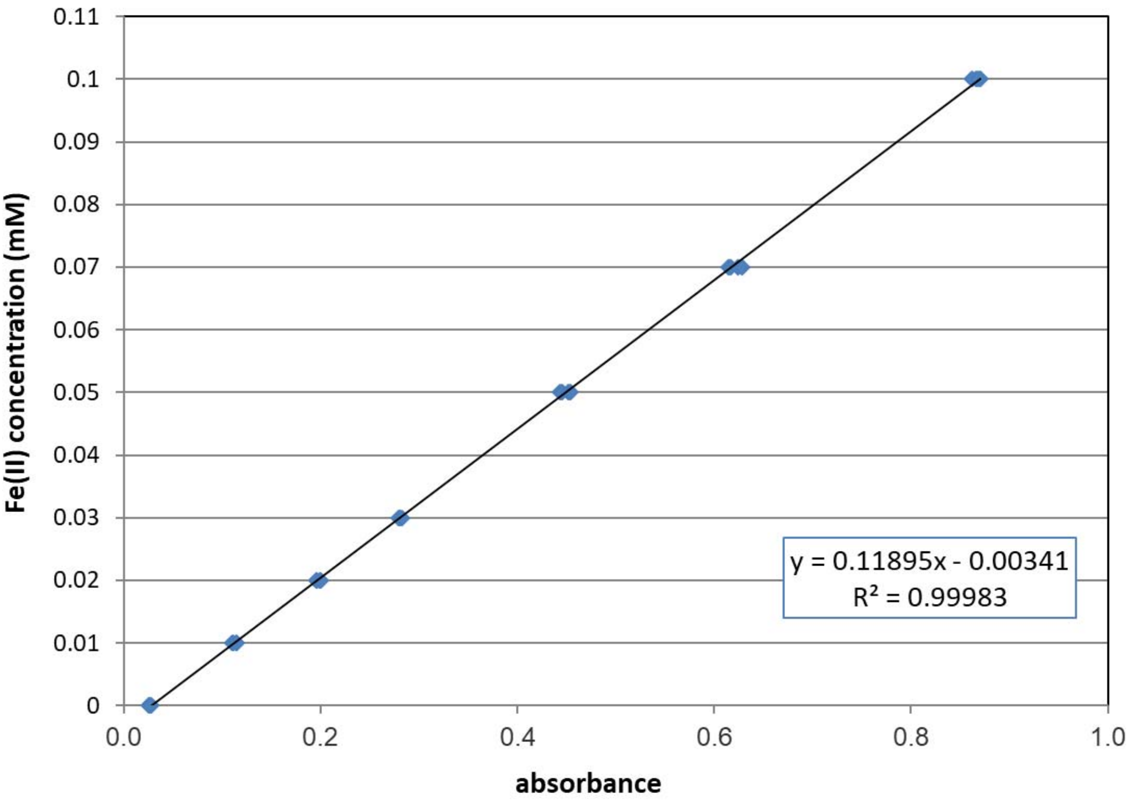

Figure S1 UV-Vis absorbance data at dissolved Fe(II) concentrations from 0 to 0.1 mM. used to derive a calibration curve. Datapoints were from from two series of calibration series, one prepared in the undiluted solution used for reductive precipitate dissolution experiments, and one prepared in the reductive dissolution solution diluted 10-fold using BPY-free MOPS-ascorbate solution, both measured immediately after preparation as well as after 2 h reaction time. The near overlap of datapoints at a given Fe(II) concentration indicated that neither the dissolution of the reductive dissolution solution nor sample storage for 2 h significantly affected the absorbance readings. From the slope of the linear regression line over the pooled data, a molar extinction coefficient of 8400 M^{-1} cm^{-1} for the Fe(II)-BPY_3 complex at 522 nm was derived. For the quantification of Fe(II) in experimental samples, the measured absorbances were corrected for the absorbance measured in blank samples (reductive dissolution solution without precipitate).

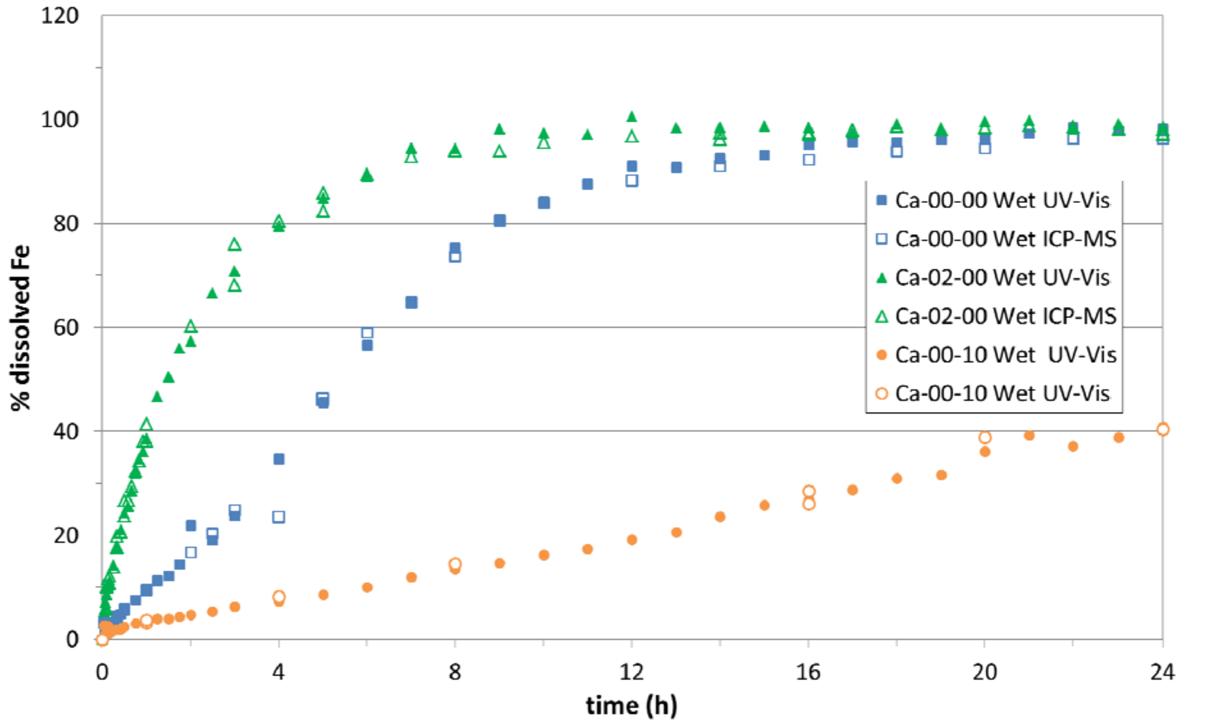

Figure S2 Comparison of UV-Vis data for dissolved Fe(II) with ICP-MS data for Fe_{tot} in 0.1-µm filtered solutions for the treatments Ca-00-00 wet, Ca-02-00 wet and Ca-00-10 wet. The close agreement suggests that Fe passing the filter membranes was dissolved Fe(II) complexed by BPY. Elevated levels of Fe_{tot} at the beginning of the experiment Ca-02-00 wet may be due to filter failure resulting in the leakage of Fe(III)-precipitate colloids.

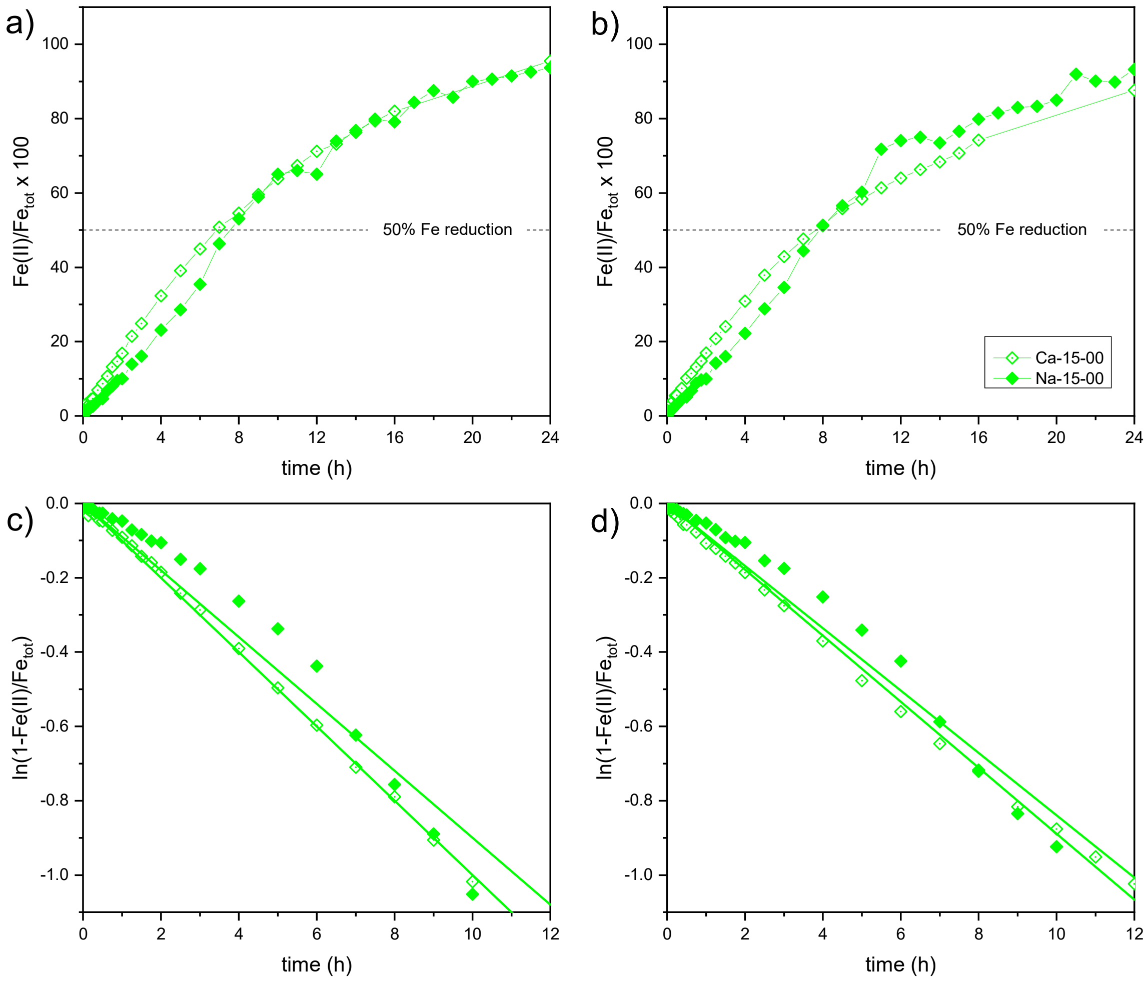

Figure S3 Dissolved Fe(II) over course of reductive precipitate dissolution for **a** wet **b** dried precipitates formed by Fe(II) oxidation at $(P/Fe)_{init}$ of 1.5 in Ca (Ca 15 00) or Na (Na 15 00) electrolyte. Thin lines serve to guide the eye. Corresponding plots of ln(1-Fe(II)/Fe_{tot}) versus time for **c** wet **d** dried precipitates. Solid lines calculated with k_{app} derived from linear regression of the experimental data (Table 1).

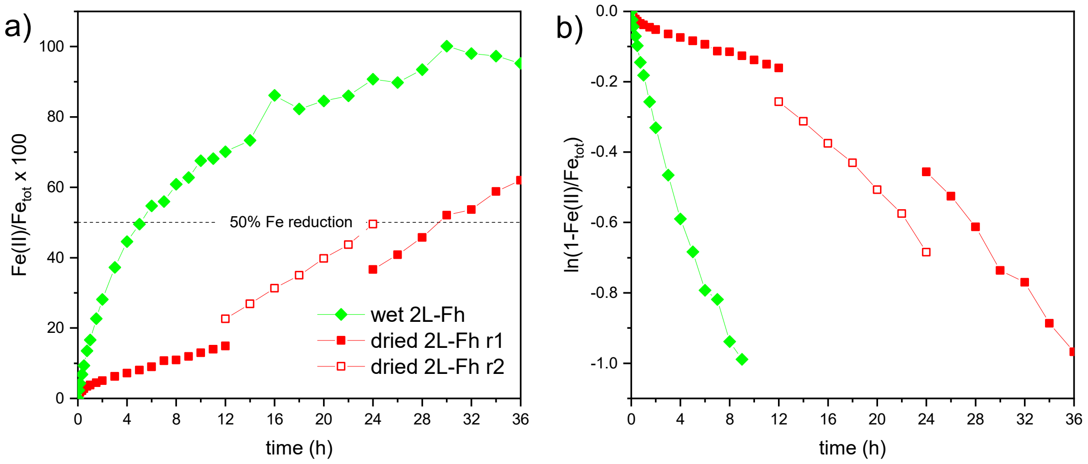

Figure S4 **a** Dissolved Fe(II) over course of reductive dissolution of wet and dried 2L-Fh and **b** corresponding plots of ln(1-Fe(II)/Fe_{tot}) versus time. For dried 2L-Fh, the results from two replicate experiments r1 and r2 are shown separately. The difference between these replicate experiments was most probably due to uncertainties in the total Fe concentrations based on which normalized concentrations were calculated (see also Table S1). The $t_{50\%}$ for dried 2L-Fh reported in Table 1 and Figure 4 corresponds to the average of the $t_{50\%}$ of the replicates.
